# Halloysite Nanotubes: Controlled Access and Release by Smart Gates

**DOI:** 10.3390/nano7080199

**Published:** 2017-07-28

**Authors:** Giuseppe Cavallaro, Anna A. Danilushkina, Vladimir G. Evtugyn, Giuseppe Lazzara, Stefana Milioto, Filippo Parisi, Elvira V. Rozhina, Rawil F. Fakhrullin

**Affiliations:** 1 Dipartimento di Fisica e Chimica, Università degli Studi di Palermo Viale delle Scienze, pad. 17, 90128 Palermo, Italy; giuseppe.cavallaro@unipa.it (G.C.); stefana.milioto@unipa.it (S.M.); filippo.parisi@unipa.it (F.P.); 2 Institute of Fundamental Medicine and Biology, Kazan Federal University, Kreml uramı 18, Kazan, 420008 Republic of Tatarstan, Russia; anchutka124@gmail.com (A.A.D.); vevtugyn@gmail.com (V.G.E.); rozhinaelvira@gmail.com (E.V.R.)

**Keywords:** halloysite, nanocomposite, cellulose, controlled release

## Abstract

Hollow halloysite nanotubes have been used as nanocontainers for loading and for the triggered release of calcium hydroxide for paper preservation. A strategy for placing end-stoppers into the tubular nanocontainer is proposed and the sustained release from the cavity is reported. The incorporation of Ca(OH)_2_ into the nanotube lumen, as demonstrated using transmission electron microscopy (TEM) imaging and Energy Dispersive X-ray (EDX) mapping, retards the carbonatation, delaying the reaction with CO_2_ gas. This effect can be further controlled by placing the end-stoppers. The obtained material is tested for paper deacidification. We prove that adding halloysite filled with Ca(OH)_2_ to paper can reduce the impact of acid exposure on both the mechanical performance and pH alteration. The end-stoppers have a double effect: they preserve the calcium hydroxide from carbonation, and they prevent from the formation of highly basic pH and trigger the response to acid exposure minimizing the pH drop-down. These features are promising for a composite nanoadditive in the smart protection of cellulose-based materials.

## 1. Introduction

Halloysite clay (HNT) is a natural and abundantly available nanoparticle formed by rolled kaolin sheets. The main deposits of HNT are from Dragon Mine and Matauri Bay, which are in Utah (USA) and Northland (New Zealand), respectively. Due to its biocompatibility [1,2] HNT was recently studied for the development of innovative nanomaterials useful for biotechnological applications, such as the controlled release of drugs [3,4,5,6], tissue engineering [7,8,9], oil recovery [10], and eco-compatible packaging [11,12,13]. Furthermore, several studies proved that HNT is a suitable catalytic support [14,15], as well as an efficient removal agent [16], because of its geometrical and surface properties (large specific area, hollow tubular shape, and tunable surface chemistry). Both the sizes and polydispersity are influenced by the HNT geological deposit [17]. Typically, the HNTs lengths range between 0.1 and 3.0 µm, while their external and inner diameters are ca. 50–200 and 15–70 nm, respectively [18]. The HNT surfaces are oppositely charged within a large pH range (between 2 and 8) because of their different chemical compositions [19]. Particularly, the internal surface consists of gibbsite octahedral sheet (Al–OH) groups with a positive surface charge, whereas the outer surface is composed of siloxane groups (Si–O–Si) with a negative electrical potential. Accordingly, the selective HNT functionalization can be easily achieved through electrostatic interactions between the nanoparticle surfaces and ionic molecules, such as polymers [20], surfactants [21,22], enzymes [23], and proteins [24]. Inorganic hybrid nanoparticles are considered suitable building blocks for nanomaterials with smart properties [25,26,27,28]. The HNT cavity is an efficient nanocontainer for the loading of chemically- and biologically-active compounds allowing the fabrication of hybrid nanomaterials with functional properties (antibacterial, antioxidant, and anti-acid) [4,29,30,31,32,33]. Interestingly, the release of the encapsulated species can be controlled under specific external stimuli dependent of the environmental conditions, such as pH or temperature [5]. A Monte Carlo model was successfully used to describe the effect of environmental variables (pH and temperature) on the transport and release of dexamethasone molecules from HNT [34]. A recent review [35] highlighted that a typical release time of water-soluble active molecules from the nanotubes is 5–10 h. It should be noted that slower release kinetics are generally needed for composite materials with antioxidant, flame-retardant, and antimicrobial properties. A time-extended release can be achieved by the HNT coating with thin polymeric layers or through the formation of tube-end stoppers [36,37,38,39]. Using dextrin as a smart end-stopper endowed a targeted release of the payload within cancer cells [39].

The functionalized HNT can be employed as a filler for biopolymeric matrices in order to generate functional bionanocomposite films with long-term activity [40,41]. The paper consolidation with perfluorinated modified HNT induced a flame-retardant effect on the cellulose [42]. Similarly, pristine HNTs provided thermal stability and flame-retardant effects on poly(propylene) [43]. 

The mechanical resistance of cellulose-based materials is significantly influenced by the degree of hydrolytic and oxidative reactions. The material deterioration depends on the environmental conditions (temperature, presence of oxygen, humidity, etc.) and it might be retarded by adding nanoparticles with specific anti-acid [44] and antibacterial [45] properties. It was demonstrated that non-aqueous dispersions of alkaline nanoparticles, such as calcium and magnesium hydroxide, are efficient deacidifying treatments for cellulose-based works [44,46,47]. Due to their high reactivity, these nanoparticles provide a stable neutral environment by rapidly turning into slight alkaline species (carbonates). Ca(OH)_2_ nanoparticles are typically stabilized in short-chain alcohol dispersions. A recent study proved that Ca-alkoxides are formed and they can hamper/delay the strengthening or consolidation effects of nanolimes [48]. In general, acid paper is a challenge and many approaches have been published and reviewed [49,50]. Industrial scale deacidification processes have been installed since the 1990s and the approach we propose in this study offers a benefit to the known technologies [49].

In this paper, we propose an innovative deacidification and consolidation treatment for paper based on HNT filled with calcium hydroxide and hydroxypropyl cellulose (HPC). The method represents an improvement of the consolidation obtained by HNT/HPC mixtures [42]. The selective loading of the alkaline molecule into the HNT cavity was investigated by using several microscopic techniques, while the kinetic release of calcium hydroxide was studied by pH and thermogravimetry measurements. The HNT modification with calcium salts (triphosphate) was explored as an original approach for the formation of tube end-stoppers, which can generate a time-extended release of the loaded calcium hydroxide and, consequently, a consolidation and deacidification for the treated paper. The acquired knowledge represents an advanced step for designing tubular alkaline nanoparticles with an extended deacidification activity towards cellulose-based materials.

## 2. Results and Discussion

### 2.1. Characterization of HNT/Ca(OH)_2_ with and without End-Stoppers

**Figure 1:**
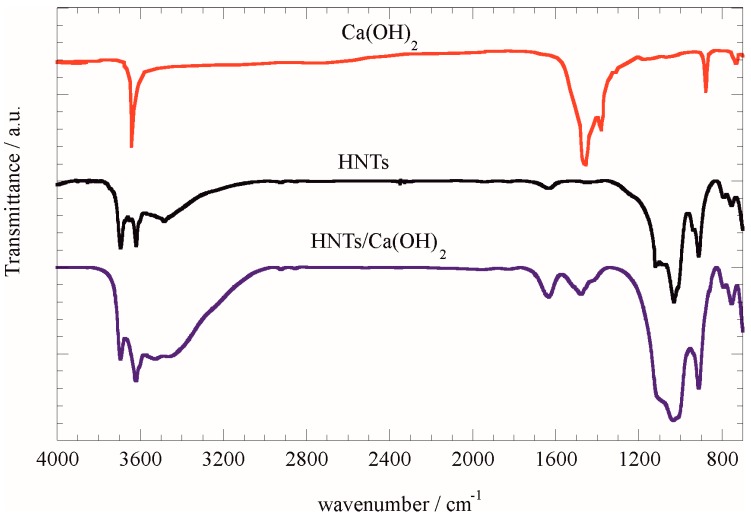
FTIR spectra for Ca(OH)_2_, HNTs and HNTs/Ca(OH)_2_.

The thermal behavior of loaded calcium hydroxide was determined by thermogravimetry. Ca(OH)_2_ presents a mass loss from 400 to 600 °C due to the dehydration process and CaO formation (Figure S1). Halloysite nanotubes present ca. 20 wt % mass loss due to hydration water [11]. By comparing the thermoanalytical curves of pristine materials and the HNT/Ca(OH)_2_ composite (Figure S1) it turned out that, in the composite material, the Ca(OH)_2_ is likely present as an additional mass loss is observed. The Ca(OH)_2_ loaded amount can be evaluated by considering the residual mass at 900 °C for pristine components and assuming the rule of mixtures. On this basis, one can calculate a value of 3.9 ± 0.2% *w*/*w* (corresponding to 4.5 ± 0.3% *v*/*v*) for the loading. Given that the full geometrical filling would provide ca. 10% *v*/*v* of loaded material [19], one may conclude that ca. half of the lumen is filled by the calcium hydroxide. The presence of Ca(OH)_2_ in the HNTs-Ca(OH)_2_ composite was confirmed by Fourier transform infrared spectroscopy (FTIR) spectra. As evidenced in Figure 1, the composite material presents the characteristic bands of both components, proving that during the loading procedure the Ca(OH)_2_ is preserved and incorporated in the composite.

**Figure 2:**
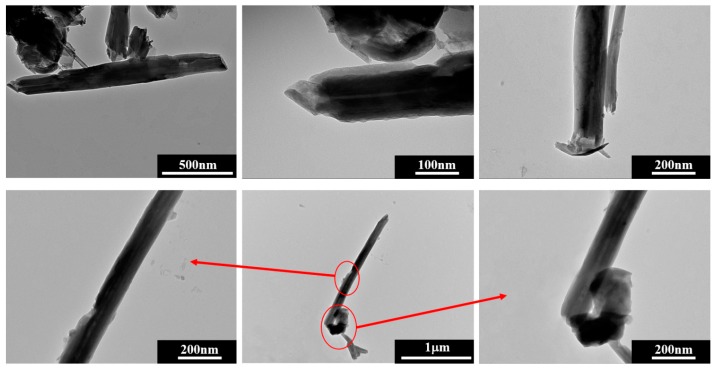
TEM images of HNTs/Ca(OH)_2_ with calcium phosphate end-stoppers.

Thermogravimetric analysis (TGA) data on HNTs-Ca(OH)_2_ with calcium phosphate end-stoppers did not show any significant difference from the HNTs-Ca(OH)_2_ sample as proof that the end-stopper treatment did not alter the general composition of the material to a large extent (Figure S1). To investigate the end-stopper formation, TEM experiments were carried out on HNTs-Ca(OH)_2_ with calcium phosphate end-stoppers. Literature reports on TEM images for HNTs samples were able to identify the lumen filling especially for high electron density materials, such as metals [51,52]. The images for HNTs-Ca(OH)_2_ based system show that the lumen of HNTs is filled (Figure 2, additional images are in Figure S2). 

**Figure 3:**
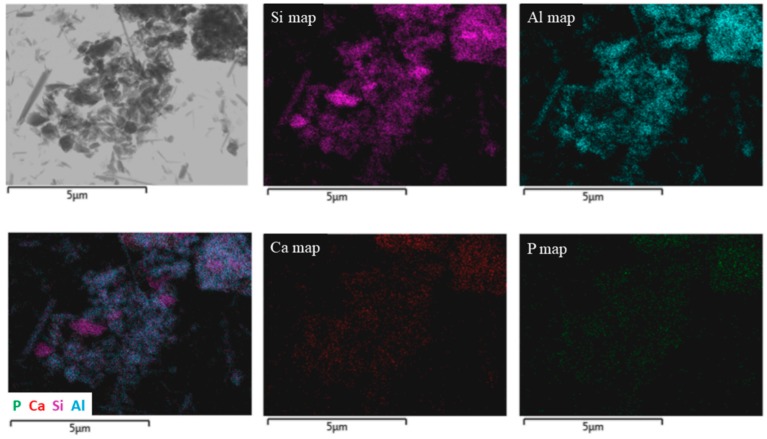
TEM image and EDX mapping of HNTs/Ca(OH)_2_ with calcium phosphate end-stoppers.

**Figure 4:**
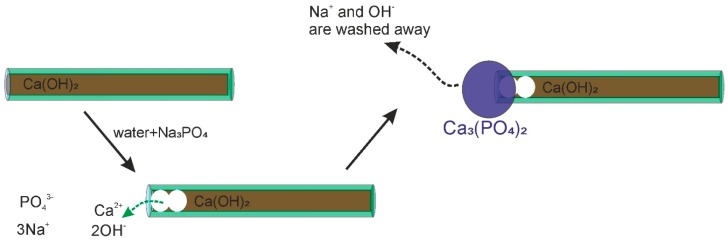
Sketch of the end-stopper formation.

EDX mapping allowed us to make a proper identification of the filling; as Figure 3 shows, the Ca signals come from the same spots where tubular-like nanostructures are imaged. As a confirmation, this is also the case for Al and Si, which are HNT components, and the Ca signal is absent in pristine HNTs. Going further, a phosphorus signal was detected, proving that phosphate was, by some means, kept in the sample during the treatment. Its concentration is relatively small and far below that stoichiometrically expected for Ca_3_(PO_4_)_2_. On the other hand, P is not phase separated within the observed sample. By a close look at the nanotube ends (Figure 1), it is revealed that they are closed by what appears to be a stopper, moreover the lumen cavity nearby the HNTs’ termination appears empty. Such a morphological observation is in agreement with a mechanism of end stopper formation based on the reaction between partially-released Ca(OH)_2_ and Na_3_PO_4_ in proximity of the nanotube ends forming Ca_3_(PO_4_)_2_ due to a high local concentration. It should be noted that a flow of Na_3_PO_4_ aqueous solution is used and that a short solution-HNT/Ca(OH)_2_ contact time is ensured by vacuum filtration in order to avoid a complete HNT unload. A schematic representation of end stoppers’ formation is depicted in Figure 4.

**Figure 5:**
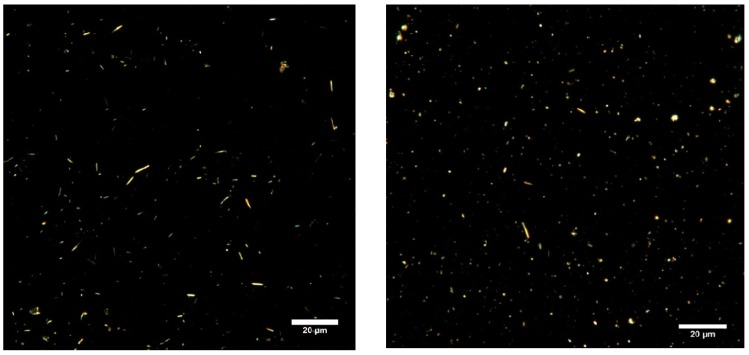
DF optical images of HNTs (**left**) and HNTs/Ca(OH)_2_ (**right**) with calcium phosphate end-stoppers in water.

Additional dark field optical images were taken from the aqueous dispersion HNTs-Ca(OH)_2_ with calcium phosphate end-stoppers. Figure 5 shows that the nanotubes generate a uniform dispersion as they are not aggregated in water. Therefore, the preparation protocols avoid any clustering of nanoparticles. The literature reports that aggregation and dispersion behaviours of halloysite nanotubes (HNTs) can be influenced by pH [53]. In particular, it is reported that the pH variation could be used as a strategy for blocking and opening the halloysite cavity. In our system, based on the observed morphology by TEM and dark field (DF) microscopy, we can exclude a clustering of HNTs-Ca(OH)_2_ nanoparticles and, therefore, the controlled access/release due to aggregative phenomena. 

### 2.2. Kinetics Study on Carbonatation and Release of Ca(OH)_2_ from HNT Lumen

**Figure 6:**
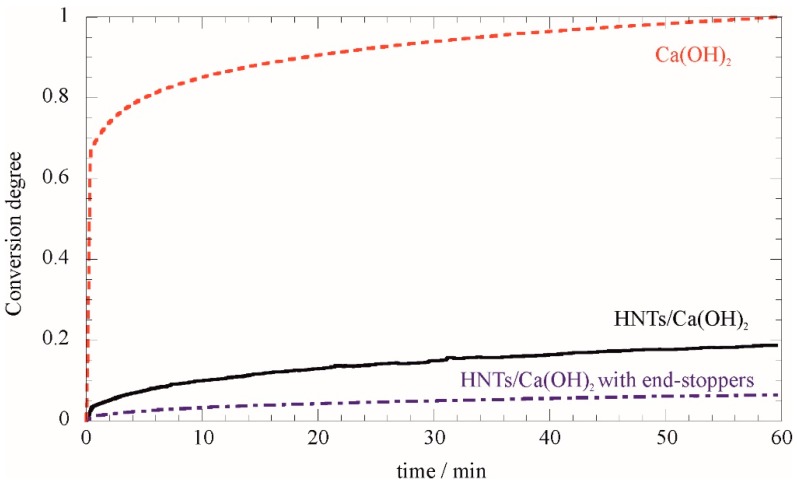
Degree of Ca(OH)_2_ carbonation in a CO_2_ atmosphere for Ca(OH)_2_, HNT/Ca(OH)_2_ and HNT/Ca(OH)_2_ with phosphate end-stoppers.

In addition to the interesting molecular architecture, we investigated the functionality of the end stopper in playing any barrier role for gas or to control the release of Ca(OH)_2_ from the lumen. Calcium hydroxide typically undergoes CO_2_ capture with CaCO_3_ formation. This process has been widely investigated due to the relevant applicative interest [54]. To explore the ability of HNT lumen in controlling such a process, we used thermogravimetric analysis under a CO_2_ atmosphere. The degree of Ca(OH)_2_ conversion to CaCO_3_, based on measured mass gain and initial Ca(OH)_2_ content in the measured sample is provided in Figure 6 as a function of time.

It is worth noting that confining Ca(OH)_2_ within the HNTs lumen cavity significantly retards the carbonation reaction. Furthermore, the end-stoppers prevent the CO_2_ contact and less than 10% of the calcium hydroxide is converted to carbonate after 1 h under the experimental conditions. Although the time frame is relatively short (one hour), the experiment proves that encapsulated Ca(OH)_2_ is still in its original form when bare Ca(OH)_2_ undergoes complete conversion. This result is very promising for applications as it shows the possibility to keep Ca(OH)_2_ preserved from carbonation during the treatment.

**Figure 7:**
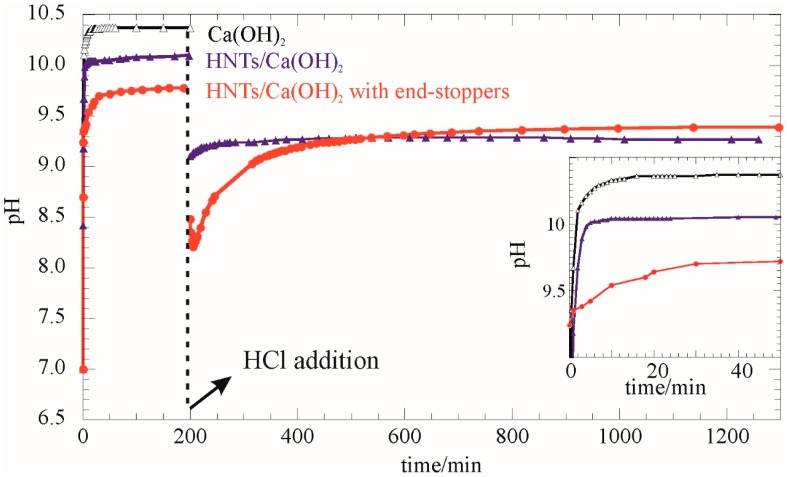
pH measurements in aqueous dispersion before and after HCl solution addition as functions of time. The inset reports an enlargement of the initial release.

The release kinetics of Ca(OH)_2_ in water were investigated by measuring the pH of the dispersion over time. To this aim, the aqueous dispersions of HNT and HNT/Ca(OH)_2_ with and without end-stoppers (0.1 wt %) were left to equilibrate under static conditions while a glassy electrode was used to monitor the pH. A blank experiment reporting the kinetics for pure Ca(OH)_2_, in the same amount as the loaded value in HNTs, revealed a quick dissolution of the hydroxide that occurs within 5 min. After that a constant pH value was approached, 0.15 cm^3^ of HCl (0.1 M) was added to the dispersion, and the pH response was measured for 18 h. With respect to the release in water, the HNT/Ca(OH)_2_ composite showed a sustained increase of pH (Figure 7). Even slower is the pH increase for the composites with the phosphate end-stoppers being the most efficient in retarding the hydroxide solubilization in water. It is reported that the dissolution kinetics of nanosized materials is influenced by the grain size due to high specific area and surface energy effects. It should be noted that even if the net Ca(OH)_2_ amount was similar for all samples, a higher pH is approached at the plateau for the HNT/Ca(OH)_2_ composite compared to the system with end-stoppers. This result reflects the ability of the end-stopping strategy to retain the hydroxide in the HNT lumen even in water media for a certain extent.

The HCl addition generates a sudden drop-down of the pH that slowly returns toward higher values due to a further release of the calcium hydroxide from the lumen. The pH increasing trend is significantly slowed by the end-stopper presence. Moreover, the ∆pH, due to the HCl addition and after equilibration, is 0.35 and 0.85 for HNT/Ca(OH)_2_ with and without end-stoppers, respectively. From the stoichiometric calculation a pH change of 1.35 is expected if all of the calcium hydroxide would have been dissolved prior to HCl addition. Therefore, we might conclude that confining Ca(OH)_2_ into the HNT lumen generates an alkaline reservoir which is released in response to acid addition.

### 2.3. Effect of HNT/Ca(OH)_2_ on Paper Deacidification and Consolidation

The efficacy of the prepared nanomaterials on paper deacidification was monitored by cycling the aging protocols and controlling the paper conditions and its damage by pH measurements and tensile experiments. 

The paper sample without a Ca(OH)_2_ basic reservoir reaches acid pH values after the first aging cycle and it remains constant, not being able to compensate for the effect of acid gas presence (Table 1). The HNTs/Ca(OH)_2_ system generated a paper alkaline pH which systematically decreases with aging approaching the value for the paper sample without the basic reservoir. Keeping in mind the strong acidic environment used in this investigation compared to the actual situations that might be experienced in the conventional conservation for books, the obtained results are already promising. On the other hand, the end-stopped system could be considered even more performant as the starting value for pH is only slightly basic and the pH change is kept within one unit even after two aging cycles when a still alkaline/neutral pH is maintained.

**Figure 8:**
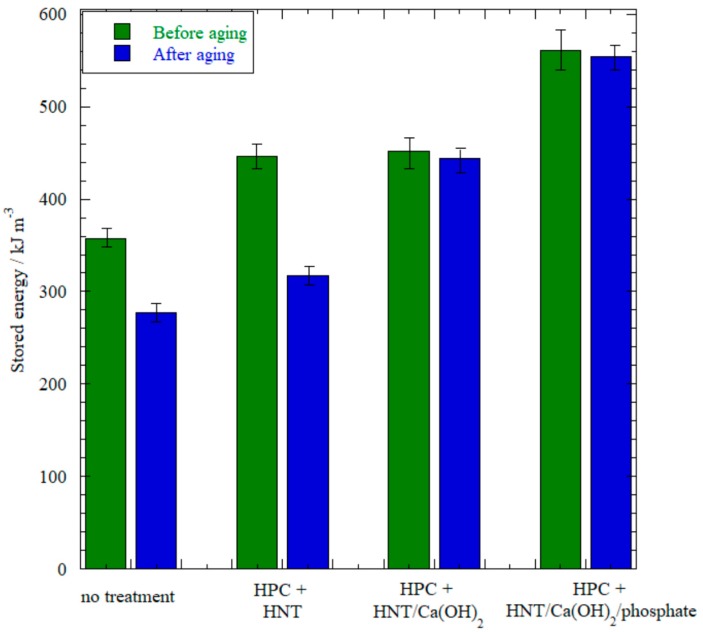
Stored energy up to sample breaking from tensile stress measurements. The error is based on the standard deviation from repeated experiments.

Tensile measurements provided information on the alteration of mechanical performance for paper samples after exposure to acidic gas. Stress at breaking point (*σ_r_*) data showed that no treatments significantly altered the paper property, while only the samples with the alkaline reservoir were able to minimize the *σ_r_* reduction upon aging (Table 1). The mechanical performance might also be described by the storage energy parameter (SE) that is obtained from the stress vs. strain curve integral and provides an idea on the maximum energy that can be adsorbed by the paper sample until it breaks down. Results in Figure 8 demonstrate that paper aging reduced the SE if the alkaline reservoir is not introduced within the paper. On the other hand, the end-stopped system is more efficient in strengthening the paper, maintaining a relatively high SE value even after the aging protocol.

## 3. Materials and Methods 

*Materials*: Halloysite nanotubes with a specific surface area of 65 m^2^·g^−1^ and a specific gravity of 2.53 g·cm^−3^ are from Sigma-Aldrich (Milan, Italy). Ca(OH)_2_, Na_3_PO_4_·12H_2_O, HNO_3_ 60%, 2-hydroxypropylcellulose (HPC), and ethanol (96%) were purchased from Sigma-Aldrich (Milan, Italy) and used without further purification. The paper sample is cellulose based from Albet^®^ (Milan, Italy) (70 g·m^−2^, thickness 0.138 mm and water capillary raise > 178 mm/h).

*Thermogravimetry analysis (TGA)*: Experiments were done using the Q5000 IR (TA Instruments, Milan, Italy) under nitrogen flow (25 cm^3^·min^−1^) by heating the samples from room temperature to 900 °C. Each sample (ca. 5 mg) was placed in a platinum pan and heated under the temperature program of 10 °C·min^−1^. Loading was calculated according to the procedure in the literature and errors were evaluated from standard deviations of three measurements [11]. The CO_2_ capturing experiments were carried out by quickly heating the sample (200 °C·min^−1^) to 600 °C in a N_2_ flow (25 cm^3^·min^−1^). Afterwards, the gas flow was switched to CO_2_ with 99.995% chemical purity (25 cm^3^·min^−1^). The mass gain was monitored for 60 min. The high temperature was chosen to accelerate the CO_2_ capture based on literature reports [55]. Calibration was carried out as reported elsewhere [56].

*Tensile Analysis*: Tensile properties on paper samples were determined by means of a DMA Q800 instrument (TA Instruments, Milan, Italy). Tensile tests were performed on rectangular paper samples (10 mm × 4 mm) under a stress ramp of 1 MPa min^−1^ at 26.0 ± 0.5 °C. We determined the stress at which the material undergoes fractures (*σ_r_*) and stored energy up to sample breaking by integrating the stress vs. strain curves. The reproducibility was checked by repeating the experiment three times.

*pH measurements*: The pH curves were obtained by using a PCD650 pH meter (Eutech Instruments, Landsmeer, The Netherlands) immersed in an aqueous dispersion of loaded nanoclay under stirring conditions. For all of the tested nanomaterials, dispersions were kept under a controlled environment, magnetic stirring, and measured continuously. Degassed water was used and the concentration of the dispersions was 0.1 wt %. The pH values of paper was measured by using a HI 1413B/50 portable pH meter with a flat-tip electrode (Hanna Instruments, Milan, Italy) in accordance with a non-destructive test that may be used to measure the hydrogen ion concentration (pH) on the surface of the paper in books and documents that constitute the collections of libraries and government archives (working procedure: TAPPI T 529 om-04).

*TEM-EDX*: For electron microscopy imaging and energy-dispersive X-ray analysis (EDX) a Hitachi HT7700 Exalens transmission electron microscope (Tokyo, Japan) was used. The samples were prepared by placing 10 µL of the suspension on a carbon-coated lace 3 mm copper grid, then dried at room temperature. TEM imaging was performed at a 100 kV accelerating voltage in TEM mode. EDX analysis was carried out in scanning transmission electron microscope (STEM) mode using an Oxford Instruments (High Wycomb, UK) X-Max™ 80T detector.

*Enhanced dark-field imaging*: During enhanced dark field microscopy experiments the images were obtained using a CytoViva^®^ enhanced dark-field condenser attached to an Olympus BX51 upright microscope equipped with fluorite 100× objectives and a DAGE CCD camera. Extra-clean dust-free Nexterion^®^ glass slides and coverslips (Schott, Mainz, Germany) were used for EDF microscopy imaging to minimise dust interference.

*Loading of Ca(OH)_2_ onto HNTs*: Degassed aqueous solution of Ca(OH)_2_ (1.5 g·dm^−3^) was mixed with halloysite powder (5 g·dm^−3^) and sonicated for 15 min. Then, the obtained suspension was stirred and kept under vacuum for 5 min resulting in light fizzling and the loaded compound condensated within the tube. This procedure was repeated three times to improve the loading efficiency. Successively, the nanotubes were separated from the aqueous phase by centrifugation and dried under vacuum at 70 °C overnight.

*End-stopper formation*: Aqueous phosphate solution was prepared by dissolving 40 g of trisodium phosphate dodecahydrate in 250 cm^3^ of water. This solution was poured onto the HNT/Ca(OH)_2_ powder placed in a Buechner funnel with filter paper placed on the perforated plate. Vacuum was applied during the pouring. The filtered material was dried by using a side-arm flask connected to a vacuum pump.

*Paper treatments*: For the paper treatment we prepared a 2 wt % HPC solution in ethanol. A certain amount of HNT (1 wt %) was added to the polymer solution and kept under stirring over night at 25 °C. The same procedure was followed by using HNT/Ca(OH)_2_ with and without end-stoppers. It should be noted that ethanol was used as the solvent to avoid calcium hydroxide solubilization during the paper treatment. The paper samples were cut in a rectangular shape (40 mm × 8 mm) and they were deeply immersed into the well-dispersed aqueous mixtures for 24 h at 20 °C. The treated samples were dried at 35 °C.

*Paper aging under acidic conditions*: Paper specimens were placed in a closed desiccator. The vapours were saturated with HNO_3_ by equilibrating the system with 30% acid solution. One aging cycle was three days. Before any characterization, the paper samples were re-equilibrated with air for 20 days.

## 4. Conclusions

We developed a novel strategy for sustained release and controlled access to the halloysite nanotubes lumen. Calcium hydroxide was loaded into the HNTs lumen and imaged by TEM and EDX mapping. End-stoppers were created when calcium hydroxide was partially released in the presence of phosphate anions. The obtained end-stoppers prevent CO_2_ from entering the tube lumen and preserving the calcium hydroxide from carbonation. Moreover, they slow the release in water, minimizing the pH jumps if an acid is added to the dispersion. These features are very promising for paper preservation, as was demonstrated by aging experiments on treated and pristine cellulose paper samples. This composite nanomaterial would allow adding an alkaline reservoir to the paper and minimizing the pH changes, as well as the aging impact on the mechanical performance of the sample. The proposed strategy could be interesting in designing and building up nanocontainers with nanogates that are sensitive to external stimuli. 

## Figures and Tables

**Table 1:** Paper pH values and stress at the breaking point before and after aging under HNO_3_ saturated vapours.

Sample	pH before Aging	pH after First Aging Cycle	pH after Second Aging Cycle	*σ_r_*/Mpa before Aging	Δ*σ_r_* ^a^/MPa
Paper	6.7	6.3	6.2	24.3 ± 0.3	−8.6
Paper + HPC/HNTs	7.7	6.2	6.3	23.7 ± 0.2	−5.0
Paper + HPC/HNTs-Ca(OH)_2_	10.4	8.5	6.2	22.8 ± 0.2	−3.3
Paper + HPC/HNTs-Ca(OH)_2_ with phosphate end-stoppers	8.5	7.6	7.6	23.6 ± 0.2	−3.2

^a^ Δ*σ_r_* represents the reduction of the stress at breaking point induced by the aging cycle.

## Supplementary Materials

The following are available online at http://www.mdpi.com/2079-4991/7/8/199/s1. Figure S1: TGA curves for HNT-based hybrid materials. Figure S2: Additional TEM figures.
