# Supplementary material for: Halloysite Nanotubes: Controlled Access and Release by Smart Gates
Source: Nanomaterials (Basel). 2017 Jul 28;7(8):199. doi: 10.3390/nano7080199 (PMC5575681; doi:10.3390/nano7080199)
Supplement: Supplementary file 1 [file nanomaterials-07-00199-s001.pdf]

Supporting Information

Halloysite Nanotubes: Controlled Access and Release by Smart Gates

Giuseppe Cavallaro,[a] Anna A. Danilushkina,[b] Vladimir G. Evtugyn,[b] Giuseppe Lazzara,[a,*] Stefana Milioto,[a] Filippo Parisi,[a] Elvira A. Rozhina,[b] Rawil F. Fakhrullin[b*]

[a]Dipartimento di Fisica e Chimica, Università degli Studi di Palermo Viale delle Scienze, pad. 17, 90128 Palermo, Italy. [b]Kazan Federal University, Kreml uramı 18, Kazan, Republic of Tatarstan 420008, Russian Federation.

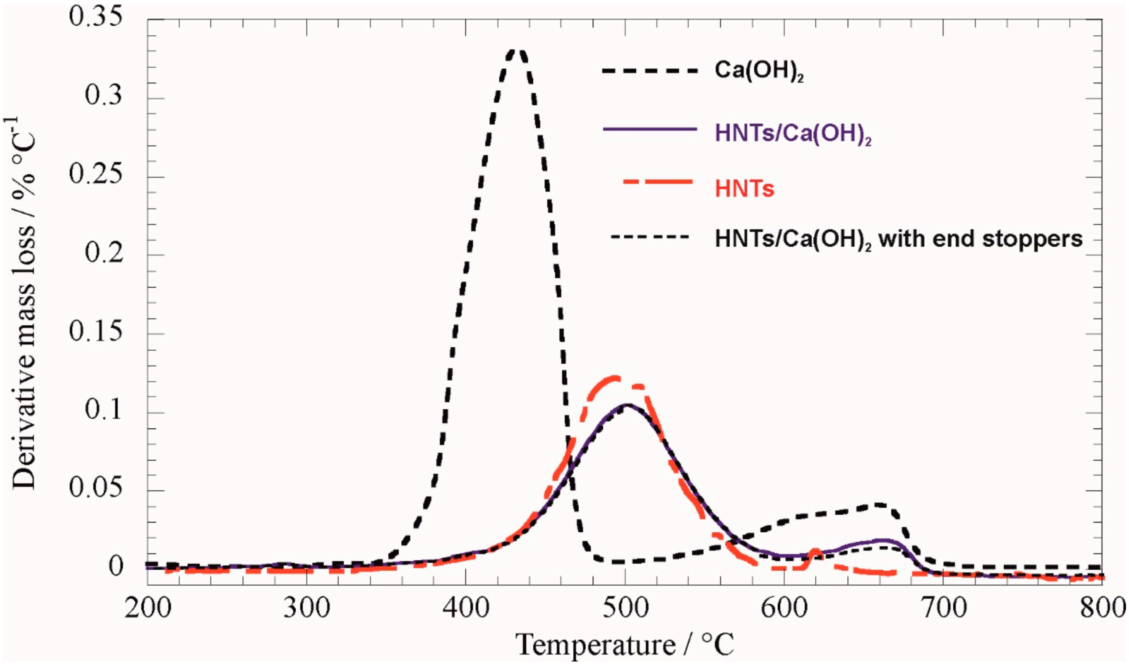

Figure S1. DTG curves for HNTs, $Ca(OH)_2$, HNTs/$Ca(OH)_2$, and HNTs/$Ca(OH)_2$ with end stoppers.

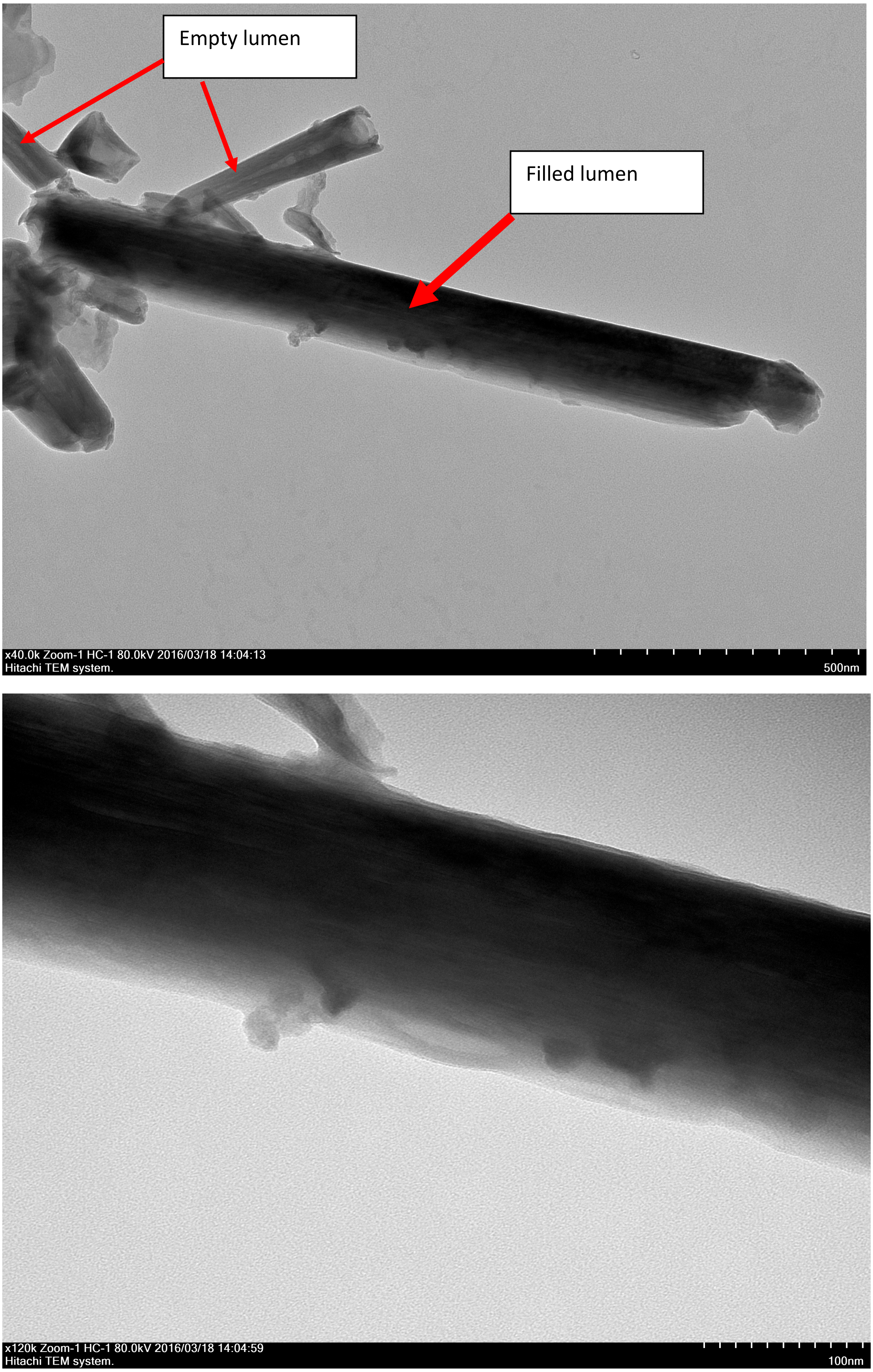

Figure S2. Additional TEM figures.
